# The challenge of separating signatures of local adaptation from those of isolation by distance and colonization history: The case of two white pines

**DOI:** 10.1002/ece3.2550

**Published:** 2016-10-27

**Authors:** Simon Nadeau, Patrick G. Meirmans, Sally N. Aitken, Kermit Ritland, Nathalie Isabel

**Affiliations:** ^1^ Natural Resources CanadaCanadian Forest ServiceLaurentian Forestry CentreQuébecQCCanada; ^2^ Department of Forest and Conservation SciencesThe University of British ColumbiaVancouverBCCanada; ^3^ Institute for Biodiversity and Ecosystem DynamicsUniversity of AmsterdamAmsterdamThe Netherlands

**Keywords:** genetic‐environment associations, isolation by colonization, isolation by environment, landscape genetics, local adaptation, *Pinus*

## Abstract

Accurately detecting signatures of local adaptation using genetic‐environment associations (GEAs) requires controlling for neutral patterns of population structure to reduce the risk of false positives. However, a high degree of collinearity between climatic gradients and neutral population structure can greatly reduce power, and the performance of GEA methods in such case is rarely evaluated in empirical studies. In this study, we attempted to disentangle the effects of local adaptation and isolation by environment (IBE) from those of isolation by distance (IBD) and isolation by colonization from glacial refugia (IBC) using range‐wide samples in two white pine species. For this, SNPs from 168 genes, including 52 candidate genes for growth and phenology, were genotyped in 133 and 61 populations of *Pinus strobus* and *P. monticola*, respectively. For *P. strobus* and using all 153 SNPs, climate (IBE) did not significantly explained among‐population variation when controlling for IBD and IBC in redundancy analyses (RDAs). However, 26 SNPs were significantly associated with climate in single‐locus GEA analyses (Bayenv2 and LFMM), suggesting that local adaptation took place in the presence of high gene flow. For *P. monticola*, we found no evidence of IBE using RDAs and weaker signatures of local adaptation using GEA and *F*_ST_ outlier tests, consistent with adaptation via phenotypic plasticity. In both species, the majority of the explained among‐population variation (69 to 96%) could not be partitioned between the effects of IBE, IBD, and IBC. GEA methods can account differently for this confounded variation, and this could explain the small overlap of SNPs detected between Bayenv2 and LFMM. Our study illustrates the inherent difficulty of taking into account neutral structure in natural populations and the importance of sampling designs that maximize climatic variation, while minimizing collinearity between climatic gradients and neutral structure.

## 1 Introduction

Climate is a major factor affecting the distribution of genetic diversity among natural populations of plants. Tree species generally exhibit moderate to high among‐population genetic variation for adaptive traits along climatic gradients (Alberto et al., 2013; Savolainen, Pyhäjärvi, & Knürr, 2007). Despite such evidence of local adaptation from common‐garden studies, patterns of population structure observed at nuclear loci are often considered to result from neutral processes affecting the whole genome, including genetic drift, gene flow, and past demographic events (e.g., recent range contractions and expansions). A more recent view is that natural selection can also affect genomewide population divergence if gene flow among ecologically divergent habitats is reduced because of selection acting against nonlocally adapted migrants (Hendry, 2004; Nosil, Vines, & Funk, 2005), or because of other nonadaptive processes (Wang & Bradburd, 2014). These processes can result in “isolation‐by‐environment” (IBE) patterns, that is, an increase in among‐population genetic differentiation with increasing environmental distance, independent of geographic distance (Wang & Bradburd, 2014; Wang & Summers, 2010). IBE has been commonly detected in natural populations of various taxa (Papadopulos et al., 2014; Sexton, Hangartner, & Hoffmann, 2014; Shafer & Wolf, 2013), including tree species (e.g., DeWoody, Trewin, & Taylor, 2015; Mosca, González‐Martínez, & Neale, 2013; Sork et al., 2010). However, whether adaptive or neutral processes, or a combination of both, have created the observed population structure remains unknown for many species.

Disentangling IBE from neutral patterns of genetic variation is challenging (Shafer & Wolf, 2013; Wang & Bradburd, 2014). For example, decreasing gene flow with increasing geographic distance due to restricted dispersal (i.e., isolation by distance, IBD; Wright, 1943) can produce patterns similar to IBE when geography is correlated with environmental variation (Meirmans, 2012; Orsini, Vanoverbeke, Swillen, Mergeay, & De Meester, 2013). Postglacial recolonization can also generate allele frequency gradients similar to IBE or IBD as a result of repeated founder events and “allele surfing” along the colonization front (de Lafontaine, Ducousso, Lefèvre, Magnanou, & Petit, 2013) because colonization routes often covary with environmental gradients. Furthermore, postglacial recolonization from different glacial refugia followed by secondary contact can also create genetic barriers (hereafter referred to as isolation by colonization, IBC) that often coincide with environmental clines (e.g., Bierne, Welch, Loire, Bonhomme, & David, 2011; Richardson, Rehfeldt, & Kim, 2009). Hence, because the selective climatic gradients, geography, and postglacial recolonization routes are often spatially correlated in natural populations, it is extremely difficult to separate the relative effects of IBE from those of IBD and IBC. However, disentangling these effects is important to accurately control for neutral population structure (e.g., IBD and IBC) when looking for signatures of local adaptation.

Loci showing signatures of selection are often detected by testing for atypically high or low among‐population genetic differentiation compared with the rest of the genome (*F*_ST_ outlier tests; Lewontin & Krakauer, 1973; Beaumont & Nichols, 1996; Beaumont & Balding, 2004; Foll & Gaggiotti, 2008; Excoffier, Hofer, & Foll, 2009), or by looking at correlations with environmental factors of interest after controlling for neutral population structure (genetic‐environment associations, GEA; Coop, Witonsky, Di Rienzo, & Pritchard, 2010; Frichot, Schoville, Bouchard, & François, 2013; Gunther & Coop, 2013). These methods show variable performances under different demographic scenarios (Excoffier et al., 2009; Frichot, Schoville, de Villemereuil, Gaggiotti, & François, 2015; Lotterhos & Whitlock, 2014, 2015; de Villemereuil, Frichot, Bazin, François, & Gaggiotti, 2014). Specifically, GEA methods have low power and high rates of false positives when environmental gradients are correlated with the main axes of neutral population structure (De Mita et al., 2013; Lotterhos & Whitlock, 2015; de Villemereuil et al., 2014). Despite the fact that GEA methods have variable performances in such scenarios, many studies only report results from a single method, and very few report the degree of collinearity between environmental gradients and geography (e.g., Lee & Mitchell‐Olds, 2011) or phylogeographic lineages (e.g., Jaramillo‐Correa et al., 2015).

The sampling design also impacts the ability to detect IBE (Wang & Bradburd, 2014), *F*_ST_ outliers, and GEAs (Lotterhos & Whitlock, 2015; Meirmans, 2015). For GEA and IBE analyses, power can be improved by sampling individuals from as many climatically variable populations as possible across the range of a species, at the cost of sampling fewer individuals per population (De Mita et al., 2013; Poncet et al., 2010; Wang & Bradburd, 2014). In addition, simulations showed that increasing the total number of sampled individuals increased the power of GEA and *F*_ST_ outlier analyses (Lotterhos & Whitlock, 2015). Statistical methods that take into account uncertainty due to small population sample sizes (e.g., Coop et al., 2010; Foll & Gaggiotti, 2008; Frichot et al., 2013) are well suited for sampling schemes that aim to maximize environmental variation by including a large number of populations in order to improve our ability to detect signatures of local adaptation in natural populations.

**Figure 1:**
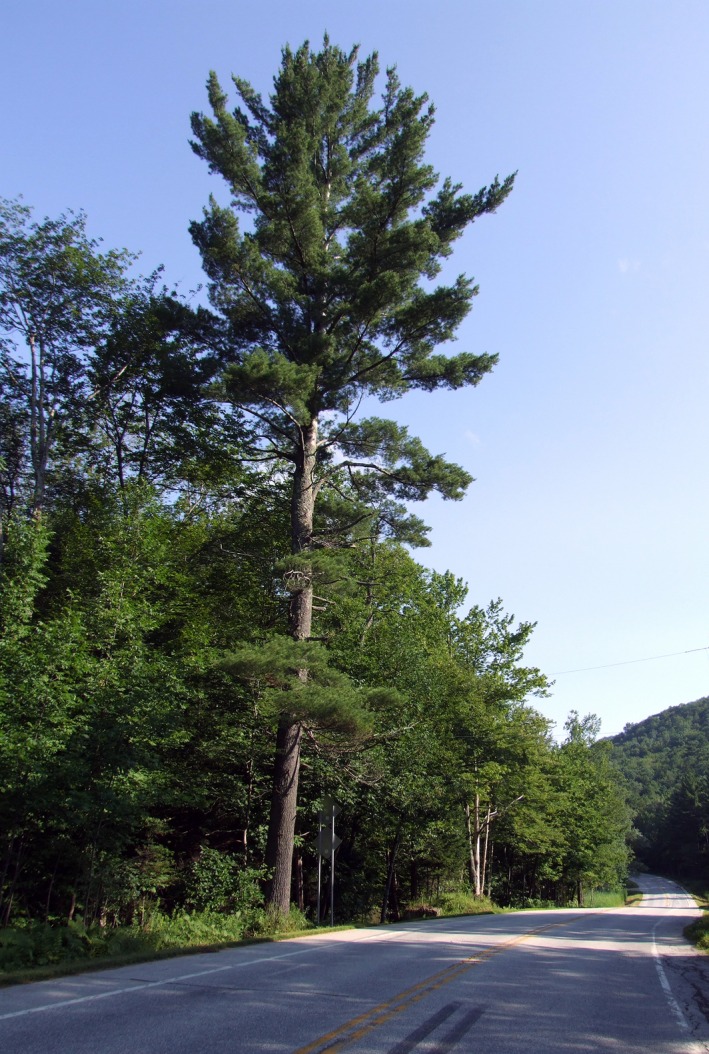
White pine tree (*Pinus strobus*) along the road (Maine, USA)

Another promising avenue to detect adaptive loci of importance is to compare signatures of adaptation among closely related species or evolutionary lineages using a set of orthologous genes (i.e., genes that descended from a common ancestral gene by speciation; e.g., Grivet et al., 2011; Mosca et al., 2012; Chen et al., 2014; Zhou, Zhang, Liu, Wu, & Savolainen, 2014). Evidence of convergent evolution (Arendt & Reznick, 2008), or the repeated evolution of similar phenotypes from similar genetic mechanisms is increasing (Stern, 2013; Jones et al., 2012; Yeaman et al., 2016), but it is currently limited to a few taxa. Eastern white pine (*Pinus strobus*, Figure 1) and western white pine (*P. monticola*) diverged <12 million years ago (Gernandt et al., 2008) and are well suited for studying local adaptation as both species are distributed latitudinally and longitudinally across a wide variety of climates in North America. However, these two species have demographic histories that could complicate the detection of signatures of local adaptation and IBE. Populations of both species cluster into southern and northern genetic groups, likely resulting from range expansion from multiple glacial refugia (Rehfeldt, Hoff, & Steinhoff, 1984; Nadeau et al., 2015; but see Richardson et al., 2009; and Zinck & Rajora, 2016; who suggested a single refugium). Differentiation between the phylogeographic groups may also be in part due to adaptation to contrasting climates as the northern and southern groups differ in their adaptive traits (e.g., height growth potential, cold hardiness; Rehfeldt et al., 1984; Richardson et al., 2009; Joyce & Rehfeldt, 2013).

Here, we look for evidence of signatures of local adaptation and IBE using single nucleotide polymorphism (SNP) markers developed from 168 orthologous genes and genotyped on 133 *P. strobus* and 61 *P. monticola* populations distributed across their natural ranges. We addressed the following questions: (1) “Can we detect genes showing signatures of local adaptation to climate in each species and in both species?” and (2) “Did local adaptation to climate contribute to the observed population structure (IBE) in *P. monticola* and *P. strobus*, or was it mostly driven by neutral processes (i.e., IBD or IBC)?”

## 2 Material and Methods

### 2.1 Sampling and SNP dataset

**Figure 2:**
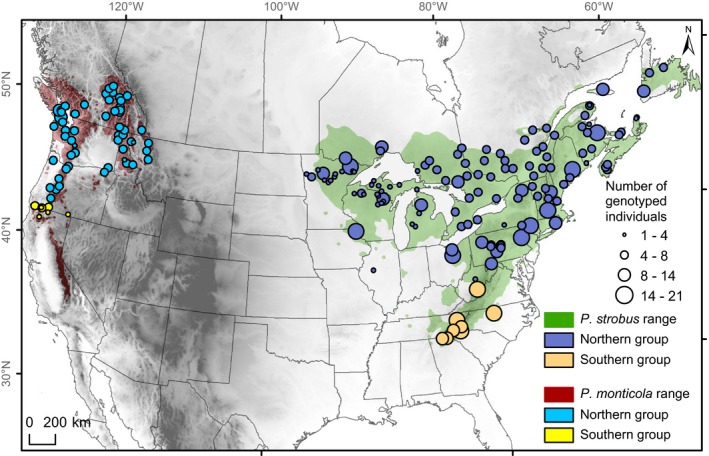
Sampling locations for *Pinus strobus* and *P. monticola*. Populations are colored according to their genetic group membership detected using STRUCTURE for *K *=* *2 (Nadeau et al., 2015)

**Figure 3:**
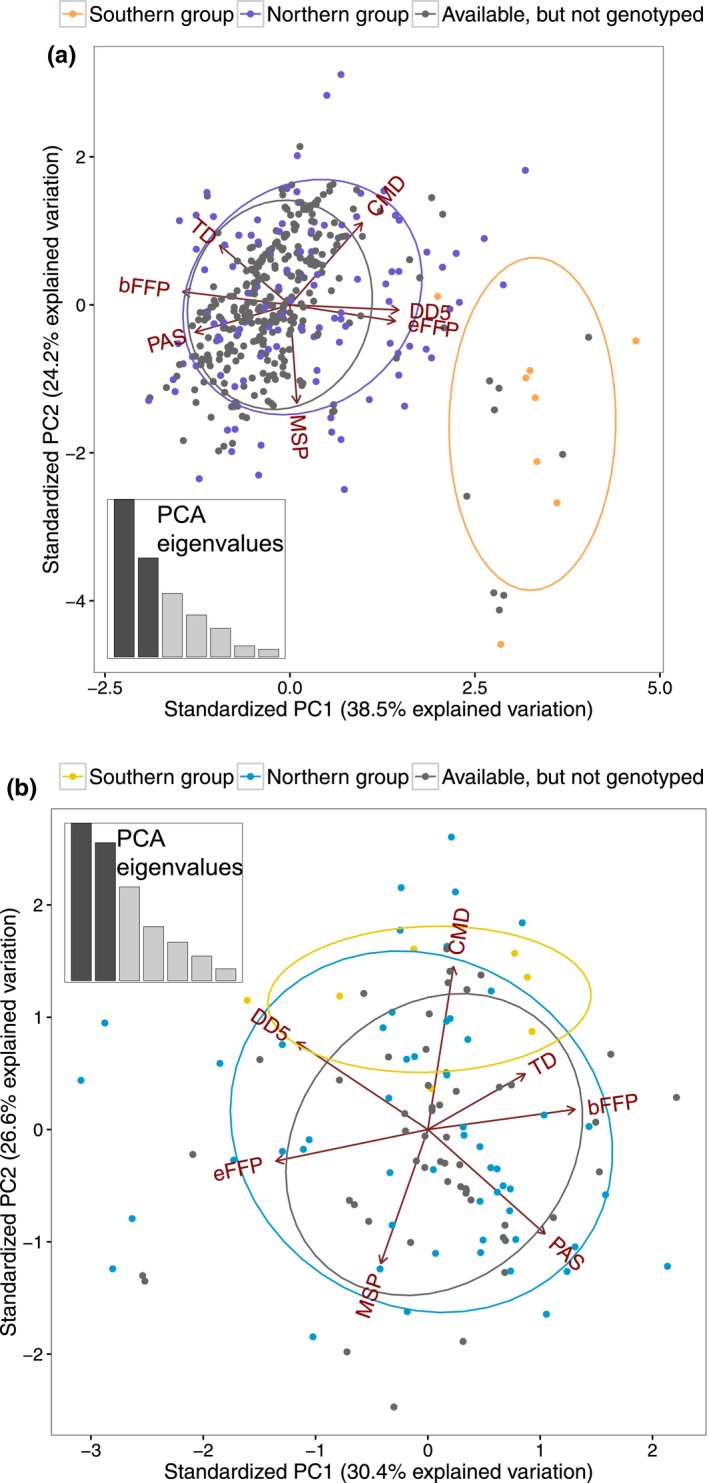
(a) *Pinus strobus* and (b) *P. monticola*: principal component analysis (PCA) including seven climatic variables obtained for available samples in seed banks and provenance trials (see Nadeau et al., 2015). Variation along PC1 (*x*‐axis) and PC2 (*y*‐axis) was used to select samples for genotyping in order to cover a wide range of environmental variation. Genotyped populations are colored according to their genetic group membership as in Figure 2. Available populations that were not genotyped (gray dots) were either not sampled or failed genotyping. Ellipses represent the 95% confidence intervals for each group. Insets show the proportion of variation explained by each PC

To investigate patterns of adaptation, we used a previously developed dataset (Nadeau et al., 2015), in which 153 (120 genes) and 158 SNPs (127 genes) were genotyped on 831 individuals (133 populations) of *P. strobus* and 348 individuals (61 populations) of *P. monticola* (Figure 2). A selection from samples available in provenance trials and seedbanks (see Nadeau et al., 2015 for details) was made to cover a large range of climatic conditions across the natural distribution of each species. To do so, we performed a principal component analysis (PCA) on seven climatic variables (see “2.2 Climatic data”; Table 1) obtained for all available samples of each species, using the prcomp function in R (R Core Team 2015), and we selected populations that covered a wide range of variation in the first two principal components (Figure 3). Note that for *P. monticola*, many provenances from southern Oregon and California were not available because they had died in the Whidbey Island provenance trial (WA, USA) before sampling.

**Table 1:** Description of climatic variables obtained for all sampled populations

Climatic variable		Units
DD5	Degree‐days above 5°C	°C
TD	Temperature difference between mean warmest month temperature and coldest month temperature, or continentality	°C
bFFP	Beginning of frost‐free period	Julian date
eFFP	End of frost‐free period	Julian date
MSP	Mean summer precipitation	mm
PAS	Precipitation as snow	mm
CMD	Hargreaves climatic moisture deficit	mm
Elev	Elevation	m

SNP development was conducted in parallel using putative orthologous gene sequences available for both *P. strobus* and *P. monticola* (i.e., sequences amplified using the same primers in both species; Nadeau et al., 2015). Briefly, an initial set of 118 gene sequences from the White Pine Resequencing Project (WHISP, http://dendrome.ucdavis.edu/wpgp; Eckert et al., 2013), randomly distributed across the genome, was selected. We also included 23 candidate genes for growth, phenology, and cold hardiness in *Picea glauca*, 24 candidate genes for wood formation in *P. glauca*, one candidate gene for adaptation to aridity in *Pinus taeda*, and two gene sequences available from GenBank (see Nadeau et al., 2015 for more details). Annotation of genes was completed from a tblastx search of the database RefSeq (http://www.ncbi.nlm.nih.gov/refseq/) using the full contigs (coding and noncoding regions). We used only those matches with *E*‐values < 1 e^−10^ to conserve only high similarity matches. To look for candidates for local adaptation, all genes were blasted (blastn, *E*‐values < 1 e^−10^) against the *Picea glauca* gene catalog (GCAT 3.3; Rigault et al., 2011), yielding a total of 52 candidate genes putatively involved in growth, phenology, and cold hardiness (El Kayal et al., 2011; Holliday, Ralph, White, Bohlmann, & Aitken, 2008; Pelgas, Bousquet, Meirmans, Ritland, & Isabel, 2011). To provide complementary information for the white pine sequences that did not had a significant blast hit on the RefSeq database, we obtained the *P. glauca* best‐ortholog annotations (GCAT 3.3 sequences are complete or near complete) from the Arabidopsis database (TAIR, https://www.arabidopsis.org/index.jsp). This was particularly useful for partial white pine sequences that were mainly composed of intron sequences.

Of 168 orthologous genes, 79 contained SNPs in both species, including 34 orthologous SNPs (i.e., occurring at the same nucleotide position in both species). Sixty‐eight *Pinus strobus* SNPs and 72 *P. monticola* SNPs occurred at different nucleotide positions within orthologous genes. Forty‐one genes (51 SNPs) and 48 genes (52 SNPs) contained SNPs only in *P. strobus* and *P. monticola*, respectively. We deduced SNP annotations (i.e., noncoding, synonymous, nonsynonymous) for 71 fully annotated genes from the WHISP dataset (Eckert et al., 2013). For the other gene sets, the *Picea glauca* gene catalog was used to deduce coding regions and SNP annotations.

### 2.2 Climatic data

Climate normals for each population for the 1961–1990 period were obtained using ClimateNA (Wang, Hamann, Spittlehouse, & Carroll, 2016). We selected seven climatic variables that did not covary strongly (*r *<* *.80) in at least one of the species (Table 1). In other words, a climatic variable that was highly correlated (*r *>* *.80) in one species could still be retained if it was less correlated in the other species (*r *<* *.80) to ensure that we did not miss any important climatic variation (Figure S1, Appendix S2). In addition, to reduce collinearity with the main axes of ancestry, we ensured that the selected variables were not highly correlated (*r *<* *.70) with the *Q*‐values from STRUCTURE (*K *=* *2 within each species) obtained from Nadeau et al. (2015). We also included elevation as a climatic surrogate (eighth climatic variable) as it represents many climatic gradients upon which selection can act and should not be strongly correlated with patterns of IBD (e.g., two populations on different mountain tops may have similar climates, but each of them is spatially closer to their warmer, lower‐lying mountain flanks than to the other mountain top). Reduction in climatic variables to principal components was avoided to make possible direct and easily interpretable comparisons between our study species.

### 2.3 *F*_ST_ outlier tests

All analyses were performed separately for each species. We first looked for signatures of selection using *F*_ST_ outlier tests. We chose to use BayeScan because it has been shown to be one of the most reliable *F*_ST_ outlier methods (De Mita et al., 2013; but see Lotterhos & Whitlock, 2014) and because it incorporates uncertainty in allele frequencies due to small population sample sizes. All simulations were performed using the default parameters, except for the prior odds (PO) for the neutral model. Increasing PO from 10 to 1,000 reduced the number of loci under balancing selection, but loci under divergent selection largely remained the same (Table S1, Appendix S1). We chose to report results with PO = 1,000 because increasing PO has been shown to reduce the number of false positives without greatly affecting the ability to detect true positives (Lotterhos & Whitlock, 2014). The internal *q*‐value function provided in BayeScan was used to assess significance, and outliers were reported at FDR <5% (*q *<* *0.05).

### 2.4 Genetic‐environment associations

Signatures of local adaptation to climate were investigated using two GEA methods that take into account neutral population structure: Bayenv2 (Coop et al., 2010; Gunther & Coop, 2013) and LFMM (Frichot et al., 2013). We first ran Bayenv2 using the entire SNP dataset and 100,000 Markov Chain Monte Carlo (MCMC) runs to estimate the covariance matrix (Figure S2, Appendix S2). We then tested associations between each SNP and each of the eight climatic variables, while including the covariance matrix as a null model, by running Bayenv2 in “test mode” with 100,000 MCMC runs. Bayes factors (BF) were averaged across 10 replicates using 10 independent estimates of the covariance matrix. The average correlation among replicates of the covariance matrix (*P. strobus*:* r *=* *.694; *P. monticola*:* r *=* *.794) and of BFs (*P. strobus*:* r *=* *.863; *P. monticola*:* r *=* *.716) were moderately high. The significance of each tested locus was determined according to Jeffrey's scale of evidence (Jeffrey, 1961): BF > 3, BF > 10, BF > 32, and BF > 100 indicated substantial, strong, very strong, and decisive support for selection, respectively.

The second GEA method used latent factor mixed models (LFMM), as implemented in the software LFMM v.1.4 (Frichot et al., 2013). This method uses a hierarchical Bayesian mixed model based on a variant of PCA, in which neutral population structure is introduced via (*k*) unobserved or latent factors. We implemented the LFMM method using the default individual‐based data specification to avoid potential biases due to unequal population sample sizes (de Villemereuil et al., 2014). To determine *k*, we performed a PCA on individual allele frequencies using the LEA package in R (Frichot & François, 2015). For each species, a Tracy–Widom test indicated that seven principal components significantly explained genetic variation (Table S2, Appendix S1), so we ran LFMM using *k *=* *7 for each species. For each test, 10,000 iterations of the Gibbs sampling algorithm were run, with the first 5,000 iterations discarded as burn‐in. *Z*‐scores from 10 independent replicate runs were combined using the Fisher–Stouffer method, and the resulting *p*‐values were adjusted using the genomic inflation factor (λ). For *k *=* *7, the average λ was close to or lower than 1 in each species (*P. monticola*: λ = 0.92; *P. strobus*: λ = 1.68) as recommended. A Benjamini and Hochberg (1995) FDR correction of 5% was applied to *p*‐values using the qvalue package in R (Storey, 2002). Figure S3 (Supporting information) shows the effect of the choice of *k* on the number of SNPs associated with each climatic variable. The overlap in outlier SNPs among analyses using different values of *k* was generally high, and the smaller overlap in *P. monticola* was mostly due to a decrease in the number of outlier SNPs with increasing *k* (Figure S4, Appendix S2).

### 2.5 IBE, IBD, and IBC

To test for IBD, we estimated the correlation between a matrix of pairwise Slatkin's linearized *F*_ST_ (*F*_ST_/(1‐ *F*_ST_)) and the matrix of log‐transformed geographic distances (Rousset, 1997) calculated using the Geographic Distance Matrix Generator online tool (http://biodiversityinformatics.amnh.org/open_source/gdmg). We then tested for IBE, while controlling for IBD, using partial Mantel tests. Climatic distances for the eight climatic variables were computed as the Euclidean distance between pairs of populations using the dist function in R. The correlation between linearized *F*_ST_ and climatic distance was tested for each climatic variable separately, with geographic distance included as a covariate. The significance of Mantel's *r* statistics for IBD and IBE was tested using *n *=* *1,000 random permutations using the mantel function in the R ecodist package (Goslee & Urban, 2007). To control for multiple testing, *p‐*values were converted into *q*‐values, and a FDR of 5% was applied based on the Benjamini and Hochberg (1995) criteria using the qvalue package in R.

A series of redundancy analyses (RDAs) were performed to partition the among‐population genetic variation into three components: IBE, IBD, and IBC. RDA is a multiple linear regression method performed between a matrix of dependent variables and matrices of independent variables. This type of multivariate analysis is more appropriate than Mantel tests when multiple climatic variables are analyzed to identify ecological drivers of population genetic structure (Orsini et al., 2013). The dependent matrix contained allele frequencies for each population. We included three independent matrices: (1) the eight climatic variables (representing IBE); (2) geographic variables (IBD); and (3) a north–south ancestry variable (IBC). For the geography matrix, we used a trend surface analysis (Borcard, Legendre, & Drapeau, 1992) to calculate second‐order polynomials and combinations of the coordinates of sampling locations (*x*,* y*,* xy*,* x* ^2^, *y* ^2^) to ensure that linear gradients in the data, as well as more complex patterns, were extracted. To prevent overfitting, we used a forward selection procedure with a stringent alpha value of 0.01 (Lee & Mitchell‐Olds, 2011). This resulted in the retention of four geographic variables for *P. monticola* (*x*,* y*,* xy*,* y* ^2^) and three for *P. strobus* (*x*,* y*,* xy*). Results were similar if we included only *x* and *y* (Table S3, Appendix S1). The north–south ancestry variable was the population *Q*‐values from STRUCTURE, which separated populations into *K *=* *2 glacial lineages (northern and southern) within each species (Nadeau et al., 2015). All three independent matrices were scaled to a mean of zero and a variance of one prior to analyses, but the dependent matrix was left untransformed. Among‐population variation in each species was partitioned into exclusive effects of climate, geography, and north–south ancestry (i.e., constrained by the effects of the remaining two independent matrices), as well as all possible combinations of these three matrices, using the varpart and rda functions of the vegan package in R (Oksanen et al., 2013). Significance of each partition was tested with the anova.cca function of vegan with a step size of 1000, resulting in at least 999 permutations.

We first performed RDAs using all SNPs to determine the main drivers of genomewide population structure. To see whether among‐population differentiation at loci under divergent selection could be explained by climate, we performed two additional sets of RDAs using subsets of SNPs detected by: 1) Bayenv2 (BF > 3); and 2) LFMM (*q *<* *0.05). Missing data in the allele frequency matrix (missing data per population; *P. monticola*: 0.2%; *P. strobus*: 0.34%) were replaced by the within‐group (northern or southern group) average allele frequency. Small sample sizes can lead to inaccurate estimates of population allele frequencies and affect Mantel tests and RDAs. Therefore, we present the results of Mantel tests and RDAs performed using populations with sample sizes ≥5 (*P. strobus*: 96 populations; *P. monticola*: 54 populations; Figure 2).

## 3 Results

### 3.1 *F*_ST_ outlier and GEA tests

We first looked for signatures of selection using *F*_ST_ outliers and GEA tests for each species separately. In *P. strobus*, BayeScan detected two SNPs that showed atypically high *F*_ST_ values (divergent selection) and three SNPs with atypically low *F*_ST_ values (balancing selection; *q *<* *0.05; Table 2). In *P. monticola*, only one SNP showed a signature of divergent selection.

**Table 2:** Number of outlier SNPs detected using BayeScan, Bayenv2, and LFMM in *Pinus strobus* and *P. monticola*. A false discovery rate of 5% was used for BayeScan and LFMM, and Bayes factor >3 was used for Bayenv2

	*P. strobus* a	*P. monticola* ^a^
BayeScan		
Divergent	2	1
Balancing	3	0
Total (%)^a^	5 (3.3)	1 (0.6)
Bayenv2 (%)^a^	12 (7.8)	12 (7.6)
LFMM (%)^a^	19 (12.4)	6 (3.8)
Total (%)^a^	29 (19.0)	18 (11.4)

^a^Numbers in parentheses indicate the proportion of outlier SNPs (number of outlier SNPs/number of SNPs tested).

**Figure 4:**
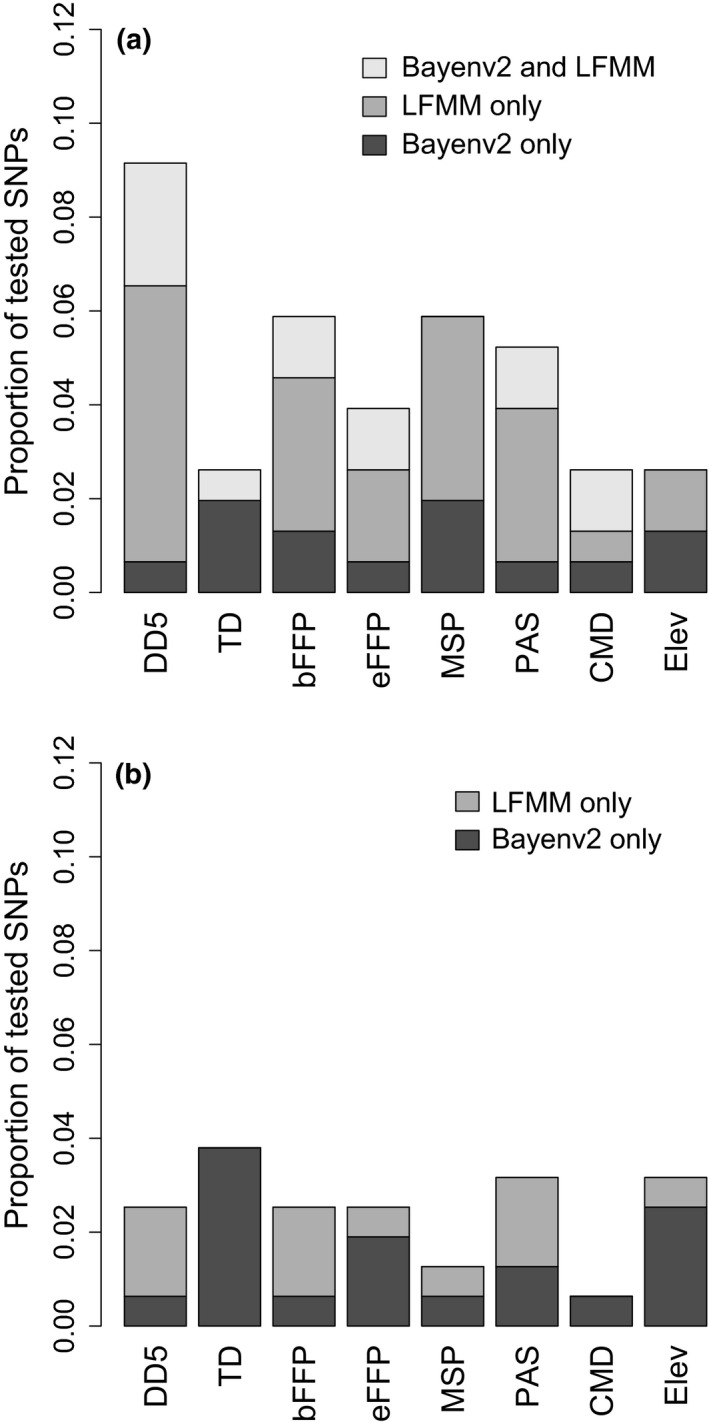
(a) *Pinus strobus* and (b) *P. monticola*: proportion of tested SNPs associated with each climatic variable by Bayenv2 (Bayes factor >3) and LFMM (*q *<* *0.05)

GEA tests (Bayenv2 and LFMM) detected several SNPs showing significant associations with one or more climatic variables (Table 2). In total, a greater proportion of SNPs was detected in GEAs for *P. strobus* than in *P. monticola* for six of the eight climatic variables tested, that is, DD5, bFFP, eFFP, MSP, PAS, and CMD (Figure 4). Top candidate SNPs in *P. strobus* had greater BFs (Bayenv2) and *Z*‐scores (LFMM) than those in *P. monticola* (Figure S5, Appendix S2). Despite the large number of SNPs detected by each method, we found little overlap between Bayenv2 and LFMM (Figure S6, Appendix S2). In *P. strobus*, five SNPs (19% of SNPs detected by GEAs) were detected by both Bayenv2 and LFMM; in *P. monticola*, no SNPs were common to both methods.

Across the *F*_ST_ outlier and two GEA methods, a total of 29 SNPs (25 genes) in *P. strobus* and 18 SNPs (18 genes) in *P. monticola* were detected as outliers by at least one method. A complete list of outlier SNPs and their annotations can be found in the online supporting information. Based on these results, we narrowed our search down to a set of highly supported candidate genes: Five SNPs (four genes) in *P. strobus* and one SNP in *P. monticola* were supported by two or more methods (Table 3). Two of these SNPs in *P. strobus* (M‐015, M‐016) were located within the same gene and were in moderate linkage disequilibrium (*r *=* *.402; Nadeau et al., 2015).

**Table 3:** Highly supported candidate SNPs, that is, detected by a minimum of two different methods. Variable names in the “Bayenv2” and “LFMM” columns refer to climatic variables that were significantly associated with the SNPs

SNP	Gene	Bayescan	Bayenv2	LFMM	SNP annotation	Putative gene function	Candidate for growth/phenology in *Picea glauca* a
*Pinus strobus*							
N‐029	0_6047_02	div***	DD5, TD, bFFP, eFFP, PAS, CMD****	DD5, bFFP, eFFP, PAS, TD, CMD, MSP, Elev****	na	basic helix‐loop‐helix (bHLH) DNA‐binding superfamily protein^c^	No
G‐014	GQ0081.BR.1_D09	div***	DD5, MSP, PAS, Elev**	DD5*	NS	Plastid movement impaired1‐related1 (PMIR1); plant‐specific C2 domain containing gene family^c^	No
M‐015	0_8683_01	ns	DD5, bFFP, PAS, CMD****	PAS, CMD, DD5, bFFP***	NS	Serine–threonine‐protein kinase at1g18390‐like^b^	Yes
M‐016	0_8683_01	ns	TD*	CMD, PAS*	NS	Serine–threonine‐protein kinase at1g18390‐like^b^	Yes
M‐017	0_8844_01	ns	eFFP, bFFP, DD5**	DD5*	Intron	Galacturonosyltransferase 13‐like^b^	Yes
*Pinus monticola*							
S‐007	CL3539‐Contig1_01	div*	Elev*	ns	Intron	TOM1‐like protein 2^b^	Yes

ns, nonsignificant; S, synonymous SNP; NS, nonsynonymous SNP; na, not annotated (no blast hit).

BayeScan and LFMM: *q <* *0.05; **q <* *0.01; ***q <* *0.001, ****q <* *0.0001. Bayenv2: *BF > 3; **BF > 10; ***BF > 32; ****BF > 100.

^a^Based on a blastn against the *P. glauca* gene catalogue (see “Materials and Methods”).

^b^RefSeq annotation; ^c^TAIR annotation of the *Picea glauca* best ortholog is provided when there was no significant hit on RefSeq.

Finally, we looked for orthologous SNPs or genes that were detected as outliers in both species by any of the three methods (BayeScan, Bayenv2, LFMM). Three of the 79 orthologous genes contained outlier SNPs in both species (Table 4). Given the number of genes that contained outlier SNPs in each species separately (*P. strobus*: 25 genes; *P. monticola*: 18 genes), the number of genes containing outlier SNPs in both species did not differ from random expectation (*p* (≥3) = .415; permutation test with 10,000 random draws in R). None of the 34 orthologous SNPs (i.e., occurring at the same nucleotide position) were outliers in both species.

**Table 4:** Genes containing outlier SNPs (any of BayeScan, Bayenv2, or LFMM) in both *Pinus monticola* and *Pinus strobus*

SNP	Gene	*P. strobus*		*P. monticola*		SNP annotation	Putative gene function (RefSeq)	Candidate for growth/phenology in *Picea glauca* a
		*F*_ST_ outlier	GEA	*F*_ST_ outlier	GEA			
T‐019	2_4724_01	ns	DD5, bFFP, eFFP*^,^ c	ns	ns	Intron	Serine–threonine‐protein kinase‐se HT1‐like	Yes
S‐021	2_4724_01	–	–	ns	bFFP, Elev*^,^ b	Intron	Serine–threonine‐protein kinase‐se HT1‐like	Yes
N‐033	0_7001_01	ns	DD5, eFFP, bFFP, PAS**^,^ c	–	–	NS	NADPH‐dependent diflavin oxidoreductase ATR3‐like isoform 2	No
P‐034	0_7001_01	–	–	ns	TD, eFFP*^,^ b	S	NADPH‐dependent diflavin oxidoreductase ATR3‐like isoform 2	No
O‐027	2_9665_01	ns	bFFP*^,^ b	–	–	NS	Interferon‐induced guanylate‐binding protein	No
Q‐032	2_9665_01	–	–	ns	PAS*^,^ c	S	Interferon‐induced guanylate‐binding protein	No

ns, nonsignificant; “–”, not tested because the SNP was not genotyped or was monomorphic in this species; S, synonymous SNP; NS, nonsynonymous SNP; na, not annotated (no blast hit).

^a^Based on a blastn against the *P. glauca* gene catalogue (see “Materials and Methods”).

^b^SNP detected by Bayenv2; *BF > 3; **BF > 10; ***BF > 32; ****BF > 100; ****BF > 100: ^c^SNP detected by LFMM: *q <* *0.05; **q <* *0.01; ***q <* *0.001, ****q <* *0.0001.

### 3.2 IBE, IBD, and IBC

#### 3.2.1 Mantel tests and RDAs using all SNPs

We investigated the importance of IBE, IBD, and IBC as drivers of genomewide population structure with partial Mantel tests and RDAs. Mantel tests detected significant IBD in both species as genetic distance increased with geographic distance (Table 5). In *P. strobus*, partial Mantel tests found that climatic distances for TD, bFFP, and eFFP were significantly correlated with genetic distances when controlled for geographic distance (*p *<* *.05), but only TD remained significant after correction for multiple testing (*q *=* *0.036). In *P. monticola*, only elevational distance significantly explained genetic distance (*p *<* *.017), and this effect was marginally significant after correction for multiple testing (*q *=* *0.077).

**Table 5:** Mantel and partial Mantel tests in *Pinus strobus* and *P. monticola*. Correlation coefficients (*r*) between (1) genetic distance (Y) and geographic distance (D); and (2) between genetic distance (Y) and each of the eight climatic variables after controlling for D

Test^a^	*P. strobus*			*P. monticola*		
	*r*	*p‐*value^b^	*q‐*value^c^	*R*	*p‐*value^b^	*q‐*value^c^
Y ~ D	.274	.001***	.014*	.339	.001***	.009**
Y ~ DD5 | D	.073	.162	.208	.081	.149	.447
Y ~ TD | D	.158	.008**	.036*	−.164	1	1
Y ~ bFFP | D	.106	.048*	.108	.017	.362	.684
Y ~ eFFP | D	.107	.032*	.096●	−.022	.608	.684
Y ~ MSP | D	.081	.133	.200	−.010	.469	.684
Y ~ PAS | D	.042	.233	.262	−.032	.573	.684
Y ~ CMD | D	−.041	.698	.698	.007	.394	.684
Y ~ Elev | D	.077	.120	.200	.223	.017*	.077●

Populations including five or more genotyped individuals were used in this analysis.

^a^Y = genetic distances calculated as the pairwise Slatkin's linearized *F*_ST_ between populations.

^b^●*p *<* *.10; *p <* *.05; **p <* *.01; ***p <* *.001.

^c^False discovery rate: ●*q *<* *0.10; *q <* *0.05; **q <* *0.01; ***q <* *0.001.

**Figure 5:**
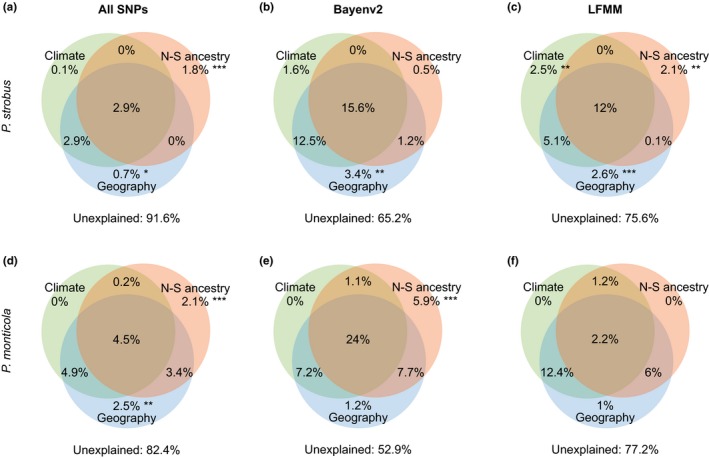
(a, b, c) *Pinus strobus* and (d, e, f) *P. monticola*: Venn diagrams showing the proportion of among‐population genetic variation explained by climate (IBE, eight climatic variables), geography (IBD,* P. monticola*:* x*,* y*,* xy*,* y* ^2^; *P. strobus*:* x*,* y*,* xy*), and north–south ancestry (IBC,* Q*‐values from STRUCTURE) in redundancy analyses (RDAs) using (a, d) all SNPs; or subsets of SNPs detected by (b, e) Bayenv2; and (c, f) LFMM. Circles in Venn diagrams are not proportional to the amount of explained variation by each factor. Significance codes: *p <* *.05; **p <* *.01; ***p <* *.001. Significance of confounded fractions between climate, geography, and north–south ancestry (overlap in circles) was not tested

Using RDAs, we partitioned among‐population genetic differentiation into three components: climate (IBE), geography (IBD), and north–south ancestry (*Q*‐values from STRUCTURE) representing recolonization history from northern and southern glacial refugia (IBC). In the uncorrected RDAs, climate, geography, and north–south ancestry each explained significant proportions of the genetic variation in both *P. strobus* and *P. monticola*, as measured by the adjusted *R* ^2^ (“combined fractions” in Table 6). A series of partial RDAs were performed to decompose their contribution to among‐population variation (“individual fractions” in Table 6; displayed as Venn diagrams in Figure 5). A total of 8.4% and 17.6% of the variation in *P. strobus* and *P. monticola*, respectively, could be explained by the three components and their various combinations (“Total explained” in Table 6). In *P. strobus*, north–south ancestry (1.8%, *p *<* *.001, constrained by climate and geography) and geography (0.7%, *p *=* *.023, constrained by climate and north–south ancestry) explained significant proportions of variation, but climate did not (0.1%, *p *=* *.382, constrained by north–south ancestry and geography). Similarly, in *P. monticola*, significant variation could be attributed exclusively to north–south ancestry (2.1%, *p *<* *.001) and to geography (2.5%, *p *=* *.006), but not to climate (0%, *p *=* *.722). For both species, 69.0 to 73.9% of the explained variation was confounded between the effects of climate, north–south ancestry, and geography (“Total confounded” in Table 6). Finally, a large portion of the variation remained unexplained (*P. strobus*: 91.9%; *P. monticola*: 82.4%). This unexplained variation could be due to environmental variables that we did not take into account (e.g., soil composition or biotic interactions), but most of it is likely due to genetic drift. For example, under a standard island model, all population differentiation is only the result of the balance between genetic drift and nonspatial migration (i.e., equal migration among all populations). In such a case, 100% of the among‐population genetic variation would remain unexplained by IBE, IBD, or IBC.

**Table 6:** Redundancy analyses (RDAs) to partition among‐population genetic variation (F) in *Pinus strobus* and *P. monticola* into three components: climate (IBE); geography (IBD); and north–south ancestry (IBC)

Combined fractions^a^	*P. strobus*						*P. monticola*					
	All (153) SNPs		Bayenv2 outlier (12) SNPs^b^		LFMM outlier (19) SNPs^b^		All (158) SNPs		Bayenv2 outlier (12) SNPs^b^		LFMM outlier (6) SNPs^b^	
	*R* ^2^	*p* (>F)^c^	*R* ^2^	*p* (>F)^c^	*R* ^2^	*p* (>F)^c^	*R* ^2^	*p* (>F)^c^	*R* ^2^	*p* (>F)^c^	*R* ^2^	*p* (>F)^c^
F~clim.	.059	.001***	.295	.001***	.193	.001***	.089	.001***	.317	.001***	.109	.002**
F~geog.	.064	.001***	.327	.001***	.198	.001***	.152	.001***	.400	.001***	.215	.001***
F~anc.	.045	.001***	.171	.001***	.139	.001***	.101	.001***	.386	.001***	.088	.001***
**Individual fractions** a												
F~clim. | (geog. + anc.)	.001	.382	.016	.091●	.025	.010**	−.006	.722	−.005	.613	−.048	.962
F~geog. | (clim. + anc.)	.007	.023*	.034	.002**	.026	.001***	.025	.006**	.012	.145	.010	.310
F~anc. | (clim. + geog.)	.018	.001***	.005	.136	.021	.002**	.021	.001***	.059	.001***	−.006	.646
F~clim.+geog. | anc.	.029	–	.125	–	.051	–	.049	–	.072	–	.124	–
F~geog.+anc. | clim.	−.001	–	.012	–	.001	–	.034	–	.077	–	.060	–
F~clim.+anc. | geog.	−.001	–	−.002	–	−.003	–	.002	–	.011	–	.012	–
F~clim. + anc. + geog.	.029	–	.156	–	.120	–	.045	–	.240	–	.022	–
Total explained^d^	.084		.348		.244		.176		.471		.228	
Total confounded^d^	.058		.293		.172		.130		.400		.218	
Total unexplained	.916		.652		.756		.824		.529		.772	
Total	1.000		1.000		1.000		1.000		1.000		1.000	

^a^F = Independent matrix of population alleles frequencies; RDA tests are of the form: F~dependent matrices | covariate matrices. Clim. = climate (eight climatic variables); geog. = geography (*P. monticola*:* x*,* y*,* xy*,* y* ^2^; *P. strobus*:* x*,* y*,* xy*); anc. = north–south ancestry (*Q*‐values from STRUCTURE). Populations including five or more genotyped individual were used in this analysis.

^b^Subsets of SNPs detected by Bayenv2 (BF > 3) and by LFMM (*q *<* *0.05). The number of SNPs for each subset is given in parentheses.

^c^●*p *<* *.10; *p <* *.05; **p <* *.01; ***p <* *.001. Significance of confounded fractions between climate, geography, and north–south ancestry was not tested.

^d^Total explained = total adjusted *R* ^2^ of individual fractions. Total confounded = Total of individual fractions confounded between various combinations of climate, geography, and north–south ancestry. Negative *R* ^2^ values were considered null for this calculation.

#### 3.2.2 RDAs using subsets of SNPs detected by Bayenv2 and LFMM

Finally, we performed RDAs using the subsets of candidate SNPs that showed signatures of local adaptation in Bayenv2 and LFMM analyses. In *P. strobus*, a significant proportion of the among‐population variation could be attributed exclusively to climate (LFMM: 2.5%, *p *=* *.010; marginally significant for Bayenv2: 1.6%, *p *=* *.091), but not in *P. monticola* (Table 6). A greater total proportion of the variation could be explained by climate, geography, north–south ancestry, and their various combinations when using SNPs detected by Bayenv or LFMM (“Total explained” in Table 6; *P. strobus*: 24.4 – 34.8%; *P. monticola*: 22.8 – 47.1%) than when using all SNPs. However, the largest proportion of this explained variation (70.5 to 96.5%) was confounded between the three components.

## 4 Discussion

In this study, we attempted to disentangle the effects of local adaptation and isolation by environment (IBE) from neutral processes, such as isolation by distance (IBD) or recolonization history from glacial refugia (isolation by colonization, IBC), in shaping among‐population genetic differentiation across the distribution of *P. monticola* and *P. strobus*. Using three GEA and *F*_ST_ outlier methods, we detected signatures of local adaptation in *P. strobus*, but such signatures were weaker in *P. monticola*. We found that, for the most part, the among‐population genetic differentiation could not be partitioned into exclusive effects of IBE, IBD, and IBC in both species, thus making it difficult to separate signatures of local adaptation from neutral patterns of population structure.

### 4.1 Signatures of local adaptation and IBE in *Pinus strobus* and *P. monticola*

Patterns of IBE occur when selection against nonlocally adapted migrants increase the genetic divergence among populations from different environments. IBE can be detected at neutral loci when divergence at selected loci extends to surrounding loci by “divergence hitchhiking” and eventually to the entire genome via “genome hitchhiking” (Feder, Egan, & Nosil, 2012; Feder & Nosil, 2010). In *P. strobus*, when using all SNPs, we did not detect strong evidence of IBE using partial Mantel tests (only TD—continentality—was significant; *q *<* *0.05), and climate did not explain among‐population variation in a RDA that controlled for IBD and IBC. However, single‐locus GEA analyses found a relatively large number of SNPs associated with climate and a significant proportion of the variation at these SNPs could be exclusively attributed to climate in multilocus RDAs. This corresponds to a scenario where gene flow is reduced among ecologically distant populations at loci directly involved in local adaptation or at closely linked loci, while there are no selective constraints on gene flow among environments for the rest of the genome (Barton, 2000; Gavrilets & Vose, 2005; Wu, 2001). This result is not surprising considering the rapid decay of linkage disequilibrium in large outcrossing populations of conifers (Namroud, Guillet‐Claude, Mackay, Isabel, & Bousquet, 2010) and the high levels of gene flow across the range of *P. strobus* (Mehes, Nkongolo, & Michael, 2009; Nadeau et al., 2015), which should uniformize among‐population genetic variation at neutral loci. Provenance trial studies have previously found moderate among‐population genetic variation for adaptive traits in *P. strobus* (e.g., height growth, bud phenology, cold hardiness; Li, Beaulieu, Daoust, & Plourde, 1997; Joyce & Sinclair, 2002; Lu, Joyce, & Sinclair, 2003a,b). Interestingly, the climatic variable “degree‐days above 5°C” was involved in the greatest number of GEAs using both Bayenv2 and LFMM, and it was also the best climatic predictor of range‐wide genetic variation in growth potential and phenology (Joyce & Rehfeldt, 2013). Thus, some of the SNPs we detected in GEAs may be important for growth potential or phenology, but confirmatory evidence would be needed from common‐garden or functional studies.

In a similar study on *P. strobus*, Rajora, Eckert, and Zinck (2016) did not detect many signatures of local adaptation in single‐locus analyses, but detected significant associations with climate using multilocus analyses with a set of 44 candidate SNPs (25 genes). In their discussion, the authors suggested that local adaptation to climate was occurring via covariance in allele frequencies among loci of small effects, rather than via allele frequency changes at a few loci of larger effects (Latta, 1998, 2004). Local adaptation via multilocus covariance is expected under high gene flow and when selection is recent (Le Corre & Kremer, 2012). These conditions are likely met in *P. strobus* since it recolonized most of its range recently, that is, following the last glacial period, and because most functional traits in conifers are expected to be controlled by a large number of genes (Hornoy, Pavy, Gérardi, Beaulieu, & Bousquet, 2015; Pelgas et al., 2011). However, high levels of gene flow can swamp divergence at weakly selected alleles and, over the long term, should favor fewer and tightly clustered alleles of large effects, depending on the amount of standing genetic variation and genetic redundancy of the trait (Yeaman, 2015; Yeaman & Whitlock, 2011). In contrast to Rajora et al. (2016), we detected a relatively large number of significant GEAs using single‐locus analyses (Bayenv2 and LFMM). *F*_ST_ outlier and GEA methods are more likely to detect moderate to strongly selected alleles because among‐population differentiation for weakly selected alleles is very difficult to distinguish from neutrally evolving loci (Le Corre & Kremer, 2012; Lotterhos & Whitlock, 2015; Yeaman, 2015). Given the small proportion of the genome surveyed here, it seems unlikely that we captured a significant amount of adaptive covariance among loci, and so we abstain from drawing conclusions about the genetic architecture of local adaptation to climate in *P. strobus*.

For *P. monticola*,* F*_ST_ outlier and GEA tests detected a smaller number of SNPs (of generally lower significance) than in *P. strobus*. Moreover, climate did not explain among‐population variation in RDAs after controlling for IBD and IBC, even for the subsets of SNPs that were detected by GEA methods. Previous studies showed no or little differentiation in phenotypic traits among populations within the large northern group, and it has been suggested that *P. monticola* has adapted to a wide variety of climates mostly via phenotypic plasticity (Chuine, Rehfeldt, & Aitken, 2006; Rehfeldt et al., 1984). For example, shoot elongation in this species is initiated later than in most temperate conifers due to a high threshold for forcing temperatures (average 10.2°C), with little genetic variation among populations (Chuine et al., 2006). Delayed shoot elongation would allow *P. monticola* to avoid late spring frost damage and to survive in a wide range of environments without the need to be locally adapted. Genetic differences for height growth potential and cold hardiness exist between the northern and southern group (Rehfeldt et al., 1984; Richardson et al., 2009). However, the small sample size for the southern group and the severe corrections for population structure applied by Bayenv2 and LFMM (Figure S5, Appendix S2) may have prevented us from separating signatures of selection from the neutral structure.

### 4.2 The role of IBE, IBD, and IBC in shaping population structure

We attempted to determine whether the genomewide population structure of both species (i.e., using all SNPs) was determined by local adaptation to climate (IBE), geography (IBD), or postglacial recolonization from glacial refugia (IBC). For both species, IBD and IBC were significant drivers of population structure, but climate alone was not. After controlling for IBE and IBD, north–south ancestry (*Q*‐values from STRUCTURE) explained the largest amount of among‐population variation in *P. strobus* and the second largest in *P. monticola*. This was expected since STRUCTURE looks for the dominant population structure patterns. For both species, populations from the northern and southern genetic groups detected by STRUCTURE may have originated from different glacial refugia, thus representing IBC (Nadeau et al., 2015), although others have suggested a single refugium (Richardson et al., 2009; Zinck & Rajora, 2016). A portion of the genetic variation included in the north–south ancestry variable could also be explained by genetic differences for adaptive traits between the northern and southern groups of each species (Joyce & Rehfeldt, 2013; Rehfeldt et al., 1984; Richardson et al., 2009). Results were similar when we did not control for IBC in RDAs: A significant proportion of the variation could be attributed exclusively to IBD, but not to IBE, and the majority of the explained variation was confounded between IBD and IBE (not shown). Thus, we were unable to determine whether local adaptation has contributed to the genetic differentiation between the northern and southern groups in either species.

Bierne et al. (2011) provide an alternative hypothesis for the existence of genetic barriers that overlap with environmental boundaries (e.g., in *P. monticola,* the north–south genetic cline coincides with contrasted environments on each side of the Cascade Mountains, Richardson et al., 2009). They argue that genetic barriers are often more likely to be maintained by endogenous barriers to gene flow resulting from environment‐independent selection such as prezygotic (e.g., mismatches in timing of reproduction) or postzygotic genetic incompatibilities among immigrants or hybrids. This is because endogenous barriers are more efficient at preventing gene flow in a larger portion of the genome than local adaptation (exogenous barrier). During glacial periods, populations surviving in separate glacial refugia can diverge via genetic drift or selection, and develop partially isolated genetic backgrounds. The endogenous barrier formed after secondary contact between two genetic backgrounds often colocates with an exogenous barrier due to the buildup of linkage disequilibrium between endogenous and exogenous loci. In summary, barriers to gene flow are often both endogenous and exogenous, and inferring the possible role of local adaptation in creating or maintaining them is very difficult (Bierne et al., 2011).

For both species, the majority of the explained among‐population variation could not be partitioned between the effects of IBE, IBD, and IBC. These spatial processes are not mutually exclusive and can act together to decrease gene flow among ecologically divergent populations (DeWoody et al., 2015; Papadopulos et al., 2014). Therefore, disentangling their relative contributions is very challenging, and attributing patterns of genetic variation to a single factor can be an oversimplification of the processes involved.

### 4.3 Comparisons between GEA methods

Bayenv2 and LFMM control for population structure in different ways. Bayenv2 first estimates a covariance matrix of allele frequencies among populations and then tests for significant genotype–environment correlations using this covariance matrix as a null model. LFMM estimates genotype–environment correlations while jointly estimating population structure via a number of latent factors (*k*, related to principal components). Although both methods essentially operate based on the same principles, that is, they test for GEAs after controlling for the portion of variation that is due to neutral population structure, their results differed greatly (*P. strobus*: 19% overlap; *P. monticola*: no overlap). The relative performance of Bayenv and LFMM depends on the demographic scenario and sampling design, and a relatively low overlap between the two methods has previously been observed in simulation studies (Lotterhos & Whitlock, 2015; de Villemereuil et al., 2014).

When the selective climatic gradients are highly collinear with neutral patterns of population structure, it becomes harder to separate neutral from selected loci, especially under weak selection (Lotterhos & Whitlock, 2015). For the subsets of SNPs detected by GEA methods in this study, the majority of the explained among‐population variation (70.5 to 95.6%) was confounded between the effects of climate, geography, and north–south ancestry, leaving only a small proportion of the variation attributed exclusively to climate (Figure 5). Depending on the underlying correction for population structure, GEA methods can attribute this confounded variation either to neutral structure (i.e., overcorrection resulting in false negatives) or to variation due to climate (i.e., undercorrection resulting in false positives). In *P. strobus*, smaller corrections due to a weaker population structure (Figures S2 and S5, Appendix S2) may explain the greater overlap between methods as compared with *P. monticola*. Thus, our results show that model differences in the correction for population structure can lead to little overlap between methods. Therefore, more studies comparing GEA methods that account differently for population structure in natural populations (e.g., multivariate RDAs controlling for geography, Lasky et al., 2012; mixed linear models controlling for kinship, Yoder et al., 2014) when adaptive patterns are correlated with demographic history are needed to better understand their relative performance.

### 4.4 Importance of the sampling strategy

The sampling design is one of the most important aspects to consider when looking for signatures of local adaptation (Meirmans, 2015). In this study, we selected a large number of populations to cover a wide range of environmental variation across the natural distribution of both species (831 individuals from 133 populations; Figure 3), and we used Bayesian programs (BayeScan, Bayenv2, and LFMM) that accounted for the uncertainty in allele frequencies due to the small population sample sizes (Coop et al., 2010; Frichot et al., 2013). Simulations showed that for a large total sample size (~900 diploids) there was little benefit in allocating individuals to more or less populations (Lotterhos & Whitlock, 2015). In a study conducted at a similar scale in *P. strobus*, Rajora et al. (2016) opted for sampling a larger number of individuals per population (22 individuals per population for SNP data) in 50 populations (total of 1100 individuals). They found only two significant SNP–environment correlations of 44 *a priori* candidate SNPs (4.5%, Spearman's rank correlation tests corrected for latitude and longitude). In comparison, our dataset included candidate genes (i.e., the 52 putative *Picea glauca* orthologs that were candidates for growth, phenology, and cold hardiness) as well as noncandidate genes. The higher percentage of GEAs detected in our study (*Pinus strobus*: Bayenv2: 9.8%; LFMM: 13.7%) may reflect the greater power derived from sampling a wider range of environmental variation. We included a larger number of populations from the southern and western edges of the distribution that experience different temperature and precipitation regimes compared with the rest of the range. Moreover, we included eight populations (104 individuals) from the southern genetic group detected in Nadeau et al. (2015), whereas Rajora et al. (2016) included only one (North Carolina). Therefore, our GEA analyses may have detected genes that are involved in local adaptation (exogenous loci) or in maintaining an endogenous barrier between the southern and northern genetic groups (Bierne et al., 2011). The different GEA methods used are also likely to account for the differences between both studies for the reasons discussed in “4.3”.

Because the effects of climate are confounded with IBD and IBC in *P. strobus* and *P. monticola*, sampling designs that minimize collinearity between environmental gradients and neutral population structure could greatly increase the power to detect signatures of local adaptation and IBE (Lotterhos & Whitlock, 2015; de Villemereuil et al., 2014; Wang & Bradburd, 2014). Sampling pairs of populations from contrasting environments, but which are relatively closely located in order to minimize differences in ancestry, showed higher power in simulations (Lotterhos & Whitlock, 2015). However, this strategy would be difficult to implement in species like *P. strobus* because climatic gradients and patterns of differentiation for adaptive traits occur at wide geographic scales, mostly along northward and westward postglacial colonization routes (Joyce & Rehfeldt, 2013; Nadeau et al., 2015; Zinck & Rajora, 2016). In such case, increasing the total number of sampled individuals (Lotterhos & Whitlock, 2015) from many ecologically different populations (including climate extremes) may be the best strategy. Analyses performed within phylogeographic genetic groups would remove the confounding effect of IBC, but may also miss some important climatic adaptation between groups. If possible, replicated transects along climatic, edaphic or phenotypic gradients that are less correlated with the main axes of neutral population structure could increase the power to detect signatures of local adaptation.

### 4.5 Highly supported candidate genes

Simulation studies showed that combining results from a number of methods can reduce false discovery rates (de Villemereuil et al., 2014) and detect loci under strong selection (Lotterhos & Whitlock, 2015). By combining the results from *F*_ST_ outlier tests and two GEA methods, we identified four and one highly supported candidate genes in *P. strobus* and *P. monticola*, respectively (Table 3). Putative orthologs of three of those genes were previously found to be important for growth and phenology in *Picea glauca* (El Kayal et al., 2011; Pelgas et al., 2011), one of which (CL3539‐Contig1_01) was included in this study for this reason (i.e., part of the 23 candidate genes for growth, phenology, and cold hardiness, see “Materials and Methods”). We detected a larger number of highly supported genes in *Pinus strobus* than in *P. monticola*. These included a serine–threonine‐protein kinase and a galacturonosyltransferase that were both found to be differentially expressed during apical bud formation in *Picea glauca* (El Kayal et al., 2011). The two remaining genes were among the strongest candidates, as they were detected by all three methods: a transcription factor (0_6047_02) involved in the differentiation of stomatal guard cells and the control of their proliferative division in *Arabidopsis* (TAIR); and a member of a plant‐specific C2 domain (GQ0081.BR.1 D09) involved in chloroplast and nuclear relocation in response to light (TAIR). Therefore, those genes are good candidates for further functional studies to confirm their role in local adaptation. SNPs that are not shared across methods should not be discarded entirely as simulations showed that loci under weak selection are often detected by only one method, although these loci may include more false positives (Lotterhos & Whitlock, 2015).

### 4.6 Overlap of outlier loci between species

Three orthologous genes showed signatures of selection in both *P. strobus* and *P. monticola* (though only detected by a single method). The discovery of genes that are involved in local adaptation to climate in both species could be expected given their relatively recent divergence (< 12 MYA) and the high degree of synteny among conserved orthologous genes in conifers (Pavy et al., 2012). One of those genes, a flavodoxin family protein (0_7001_01), was associated with the end of the frost‐free period (eFFP) and temperature‐related variables (DD5, TD) in both species. These climatic variables could be important drivers of local adaptation as phenotypic traits such as timing of budburst, timing of budset (or growth initiation and cessation), and cold hardiness vary significantly among populations of both species (Joyce & Rehfeldt, 2013; Joyce & Sinclair, 2002; Li et al., 1997; Lu et al., 2003a,b; Rehfeldt et al., 1984). The putative ortholog (i.e., gene amplified using the same primers as those used in this study) was also detected as an *F*_ST_ outlier among environmental groups defined based on DD5 in June and precipitation in December in *Larix decidua* (Mosca et al., 2013), and associated with spring–fall precipitation and aridity in *P. taeda* (Eckert et al., 2010). Thus, this gene may have evolved in response to climatic constraints in multiple tree species.

Overall, we found that the number of genes carrying outlier SNPs in both species did not differ from random expectations and that the majority of outliers were species specific. In similar comparisons made between *Picea mariana* and *P. glauca* (divergence time >10 MYA), Prunier, Laroche, Beaulieu, and Bousquet (2011) found more adaptive similarities at the gene family level (paralogs) than at the gene level (orthologs). The redundancy of functions among recently duplicated genes in conifers could have offered the possibility for selection to act on paralogous genes in different species (Namroud et al., 2010). In distantly related *P. glauca* and *Pinus contorta* (divergence time ~140 MYA), an exome‐wide study detected 47 genes (~10–18% of top candidate genes) with convergent signatures of local adaptation to low temperatures (Yeaman et al., 2016). Paralogous genes in either species were more likely to show strong signatures of convergence than one‐to‐one orthologs. Yeaman et al. (2016) sidestepped the problem of overadjustment for population structure by using uncorrected genotype–environment and genotype–phenotype correlations in each species separately to identify top candidate genes. Then, they looked for enrichment of signatures of local adaptation between both species under the assumption that genetic drift is unlikely to affect the same genes similarly between distantly related species and give rise to the same false positives. In cases where patterns of adaptation covary with neutral population structure, this method is more powerful to identify convergent loci. However, it could not be used in the current study due to the relatively modest number of loci.

## 5 Conclusions and Perspectives

In this study, we attempted to disentangle signatures of local adaptation and IBE from those of IBD and IBC in two white pine species with different demographic histories (Nadeau et al., 2015). *P. strobus* could be considered an ideal species in which to look for signatures of local adaptation since it shows moderate among‐population genetic variation for adaptive traits, but weak neutral population structure. We found in both species that a large amount of the explained among‐population genetic variation was confounded between the effects of climate (IBE), IBD, and IBC, with only a small proportion of the variation attributed exclusively to climate in *P. strobus*. Such confounding of patterns of local adaptation with neutral population structure is expected to be common in natural landscapes (e.g., Lasky et al., 2012; Lee & Mitchell‐Olds, 2011; Sork et al., 2010). Two main reasons can explain these patterns: (1) selective constraints are often spatially correlated with demographic history (e.g., northward postglacial colonization along climatic gradients); and (2) natural selection and neutral processes can act simultaneously to shape genetic variation and gene flow among populations. In this study, controlling for the putative neutral population structure resulted in very little amount of variation left to detect signatures of local adaptation and IBE.

The sampling design is typically one of the most neglected aspects in genomic studies, which often focus on the number of genetic markers, sometimes at the expense of the number of individuals and populations sampled. Sampling designs that maximize environmental variation and minimize collinearity with patterns of IBD and postglacial colonization history could greatly increase the power to detect signatures of local adaptation, while reducing the number of false positives (De Mita et al., 2013; Frichot et al., 2015; Lotterhos & Whitlock, 2015; de Villemereuil et al., 2014). Because collinearity between climate, geography, and postglacial colonization history can affect the performance of GEA methods, authors should report such correlations.

GEA methods can account differently for the confounded variation between the effects of climate, IBD, and IBC and, consequently, overcorrect or undercorrect for population structure. Combining results across a number of different methods should be standard practice to detect strong candidate genes (De Mita et al., 2013; Lotterhos & Whitlock, 2014, 2015; de Villemereuil et al., 2014). The integration of phenotypic and genotypic information from populations growing in common‐garden experiments might be the most informative approach to discover loci important for local adaptation (Sork et al., 2013; Yeaman et al., 2016), and it could be used to validate candidate SNPs detected by *F*_ST_ outlier or GEA methods (e.g., De Kort et al., 2014; Jaramillo‐Correa et al., 2015; Yoder et al., 2014). Another promising avenue would be taking advantage of the annual tree rings to establish relationships between annual growth and climatic variation in common‐garden experiments over a number of years. A genotype–phenotype association study using variation in growth responses to climate among genotypes is underway to validate the outlier loci found in this study.

## Conflict of Interest

None declared.

## Data Accessibility

Data repository (available upon request) contains GenBank accession numbers for DNA sequences (DNA sequences for the candidate genes for wood formation (J. Beaulieu, unpublished data) are available upon request); gene and SNP annotations; SNP genotype table; sampling locations and climatic data; input files and scripts for the Bayescan, Bayenv2, LFMM, and RDA analyses.
