# Supplementary material for: The challenge of separating signatures of local adaptation from those of isolation by distance and colonization history: The case of two white pines
Source: Ecol Evol. 2016 Oct 27;6(24):8649–64. doi: 10.1002/ece3.2550 (PMC5192886; doi:10.1002/ece3.2550)
Supplement: Supplementary file 1 [file ECE3-6-8649-s001.docx]

**Appendix 1 – Supplementary Tables**

**Table S1.** Number of *F*_ST_ outlier SNPs detected by BayeScan when varying the prior odds (PO) setting from 10 to 1000 in *Pinus strobus* and *P. monticola* (*q* < 0.05).

|  | *P. strobus* | *P. monticola* |
| --- | --- | --- |
| Bayescan |  |  |
| Divergent |  |  |
| PO 10 | 2 | 2 |
| PO 100 | 2 | 1 |
| PO 1000 | 2 | 1 |
|  |  |  |
| Balancing |  |  |
| PO 10 | 52 | 6 |
| PO 100 | 9 | 2 |
| PO 1000 | 3 | 0 |
|  |  |  |
| **Total** |  |  |
| PO 10 | 54 | 8 |
| PO 100 | 11 | 3 |
| PO 1000 | 5 | 1 |

**Table S2.** Principal component analysis on allele frequencies in *Pinus strobus* and *P. monticola*.

| *k* |  | *P. strobus* | |  | *P. monticola* | |
| --- | --- | --- | --- | --- | --- | --- |
|  |  | % explained variation | *p*-value^a^ |  | % explained variation | *p*-value^a^ |
| PC1 |  | 0.0217 | 8.00e-09 *** |  | 0.0543 | 8.00e-09 *** |
| PC2 |  | 0.0185 | 2.60e-04 *** |  | 0.0258 | 8.00e-09 *** |
| PC3 |  | 0.0174 | 0.019 * |  | 0.0230 | 1.24e-07 *** |
| PC4 |  | 0.0172 | 0.012 * |  | 0.0207 | 1.52e-03 ** |
| PC5 |  | 0.0168 | 0.017 * |  | 0.0196 | 0.026 * |
| PC6 |  | 0.0164 | 0.019 * |  | 0.0191 | 0.023 * |
| PC7 |  | 0.0160 | 0.039 * |  | 0.0185 | 0.045 * |
| PC8 |  | 0.0154 | 0.133 |  | 0.0180 | 0.067 |
| PC9 |  | 0.0147 | 0.594 |  | 0.0166 | 0.747 |
| PC10 |  | 0.0147 | 0.388 |  | 0.0165 | 0.561 |

^a^Tracy-Widom test: * = *p* < 0.05; ** = *p* < 0.01; *** = *p* < 0.001.

**Table S3.** Redundancy analysis (RDA) that included only latitude and longitude in the geography matrix. RDA was used to partition among-population variation (allele frequencies ,F) into three components: 1) climate (IBE); 2) geography (IBD); and 3) north-south ancestry (IBC) in *Pinus strobus* and *P. monticola*. Proportions of the variation that were exclusively attributed to climate, geography, or ancestry are highlighted in light grey. The individual fractions of the variation that were confounded between various combinations of these three components are highlighted in dark grey.

|  | *P. strobus* | | | | | |  | *P. monticola* | | | | | |
| --- | --- | --- | --- | --- | --- | --- | --- | --- | --- | --- | --- | --- | --- |
|  | All (153) SNPs | | Bayenv2 outlier (12) SNPs^b^ | | LFMM outlier (19) SNPs^b^ | |  | All (158) SNPs | | Bayenv2 outlier (12) SNPs^b^ | | LFMM outlier (6) SNPs^b^ | |
| Combined fractions^a^ | R^2^ | *p* (>F)^c^ | R^2^ | *p* (>F)^c^ | R^2^ | *p* (>F)^c^ |  | R^2^ | *p* (>F)^c^ | R^2^ | *p* (>F)^c^ | R^2^ | *p* (>F)^c^ |
| F~clim. | 0.059 | 0.001 *** | 0.295 | 0.001 *** | 0.193 | 0.001 *** |  | 0.089 | 0.001 *** | 0.317 | 0.001 *** | 0.109 | 0.004 ** |
| F~geog. | 0.058 | 0.001 *** | 0.305 | 0.001 *** | 0.185 | 0.001 *** |  | 0.083 | 0.001 *** | 0.298 | 0.001 *** | 0.064 | 0.002 ** |
| F~anc. | 0.045 | 0.001 *** | 0.171 | 0.001 *** | 0.139 | 0.001 *** |  | 0.101 | 0.001 *** | 0.386 | 0.001 *** | 0.088 | 0.001 *** |
|  |  |  |  |  |  |  |  |  |  |  |  |  |  |
| Individual fractions^a^ |  |  |  |  |  |  |  |  |  |  |  |  |  |
| F~clim. \| (geog. + anc.) | 0.003 | 0.263 | 0.016 | 0.08 ● | 0.024 | 0.015 * |  | 0.000 | 0.475 | 0.019 | 0.121 | -0.022 | 0.763 |
| F~geog. \| (clim. + anc.) | 0.004 | 0.084 ● | 0.023 | 0.003 ** | 0.018 | 0.007 ** |  | 0.004 | 0.203 | 0.010 | 0.099 ● | -0.017 | 0.882 |
| F~anc. \| (clim. + geog.) | 0.017 | 0.001 *** | 0.017 | 0.003 ** | 0.023 | 0.001 *** |  | 0.042 | 0.001 *** | 0.099 | 0.001 *** | 0.018 | 0.088 ● |
| F~clim.+geog. \| anc. | 0.028 |  | 0.126 |  | 0.053 |  |  | 0.042 |  | 0.047 |  | 0.097 |  |
| F~geog.+anc. \| clim. | -0.001 |  | 0.000 |  | -0.001 |  |  | 0.012 |  | 0.036 |  | 0.036 |  |
| F~clim.+anc. \| geog. | 0.001 |  | -0.003 |  | 0.001 |  |  | 0.022 |  | 0.046 |  | 0.086 |  |
| F~clim. + anc. + geog. | 0.027 |  | 0.157 |  | 0.116 |  |  | 0.024 |  | 0.204 |  | -0.052 |  |
| **Total explained^d^** | **0.080** |  | **0.339** |  | **0.233** |  |  | **0.148** |  | **0.463** |  | **0.237** |  |
| Total confounded^d^ | 0.056 |  | 0.283 |  | 0.169 |  |  | 0.101 |  | 0.334 |  | 0.219 |  |
| **Total unexplained** | **0.920** |  | **0.661** |  | **0.767** |  |  | **0.852** |  | **0.537** |  | **0.763** |  |
| **Total** | **1.000** |  | **1.000** |  | **1.000** |  |  | **1.000** |  | **1.000** |  | **1.000** |  |

^a^F = Independent matrix of population alleles frequencies; RDA tests are of the form: F~dependent matrices | covariate matrices. Clim. = climate (eight climatic variables); geog. = geography (*x* and *y*); anc. = north-south ancestry (Q-values from STRUCTURE). Populations including five or more genotyped individual were used in this analysis.

^b^Subsets of SNPs detected by Bayenv2 (BF > 3) and by LFMM (*q* < 0.05). The number of SNPs for each subset is given in parentheses.

^c^● = *p* < 0.10; * = *p* < 0.05; ** = *p* < 0.01; *** = *p* < 0.001. Significance of confounded fractions between climate, geography, or north-south ancestry (dark grey rows) was not tested.

^d^Total explained = total adjusted R^2^ of individual fractions (light grey + dark grey rows). Total confounded = Total of individual fractions confounded between climate, geography, or north-south ancestry (dark grey rows). Negative R^2^ values were considered null for this calculation.
