# Supplementary material for: The challenge of separating signatures of local adaptation from those of isolation by distance and colonization history: The case of two white pines
Source: Ecol Evol. 2016 Oct 27;6(24):8649–64. doi: 10.1002/ece3.2550 (PMC5192886; doi:10.1002/ece3.2550)
Supplement: Supplementary file 2 [file ECE3-6-8649-s002.docx]

**Appendix 2 – Supplementary Figures**


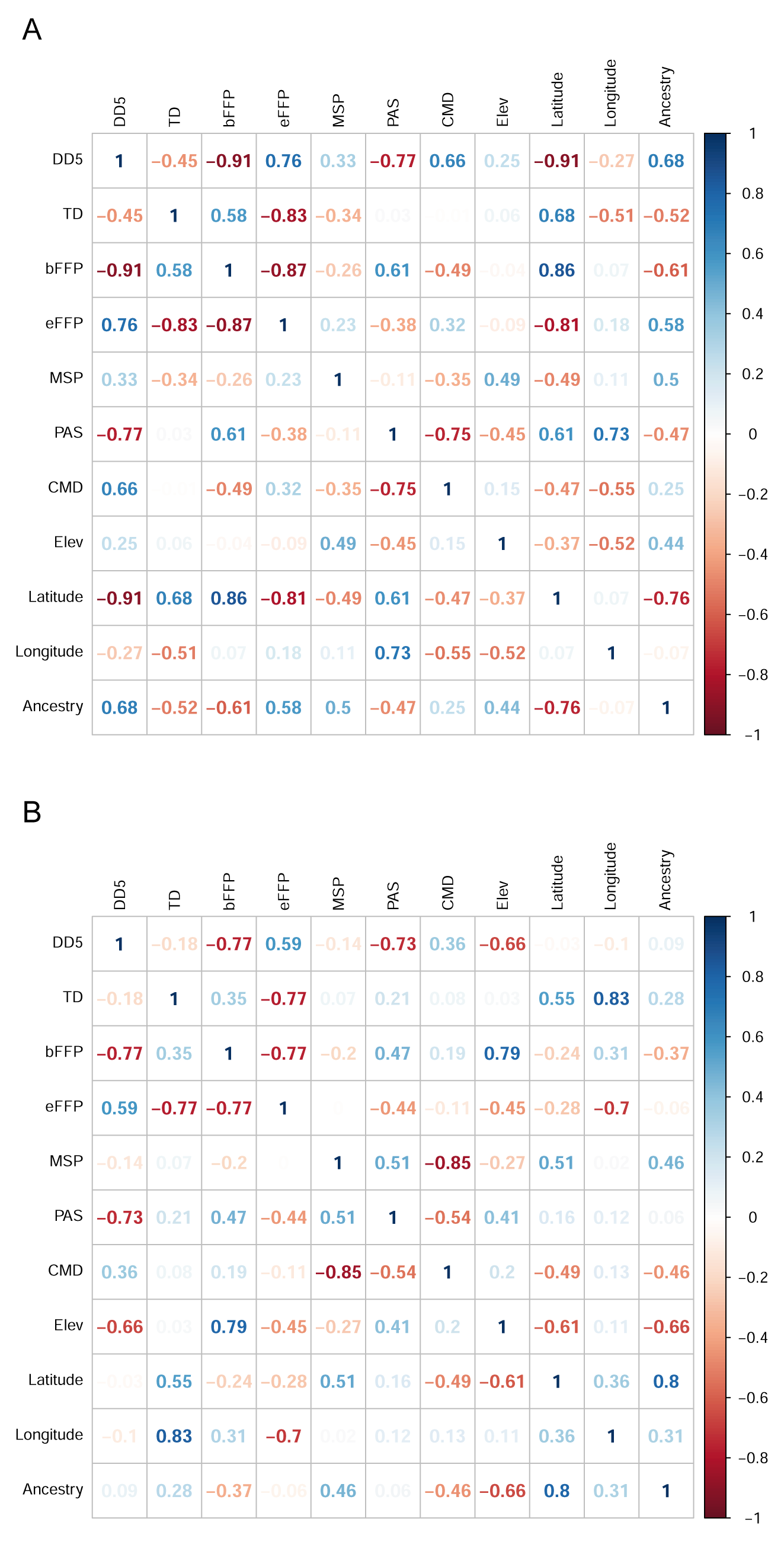


**Figure S1.** (A) *Pinus strobus* and (B) *P. monticola*: correlation matrix among the selected climatic variables, the geographic variables (latitude, longitude), and the north-south ancestry coefficients (*Q*-values from STRUCTURE for *K* = 2).


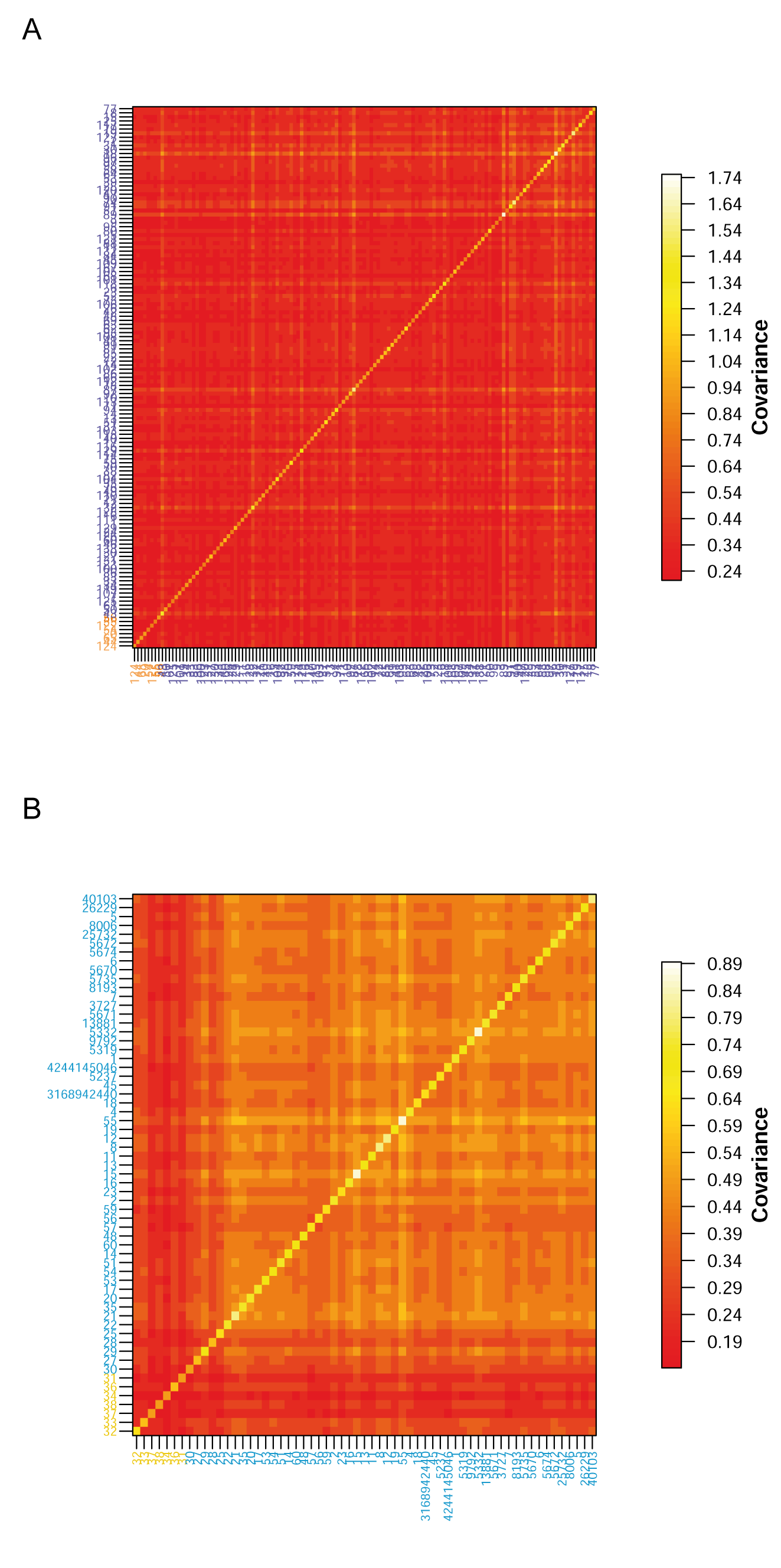


**Figure S2.** (A) *Pinus strobus* and (B) *P. monticola*: covariance matrices from Bayenv2. Populations labels are colored according to their genetic group membership as in Fig. 2.


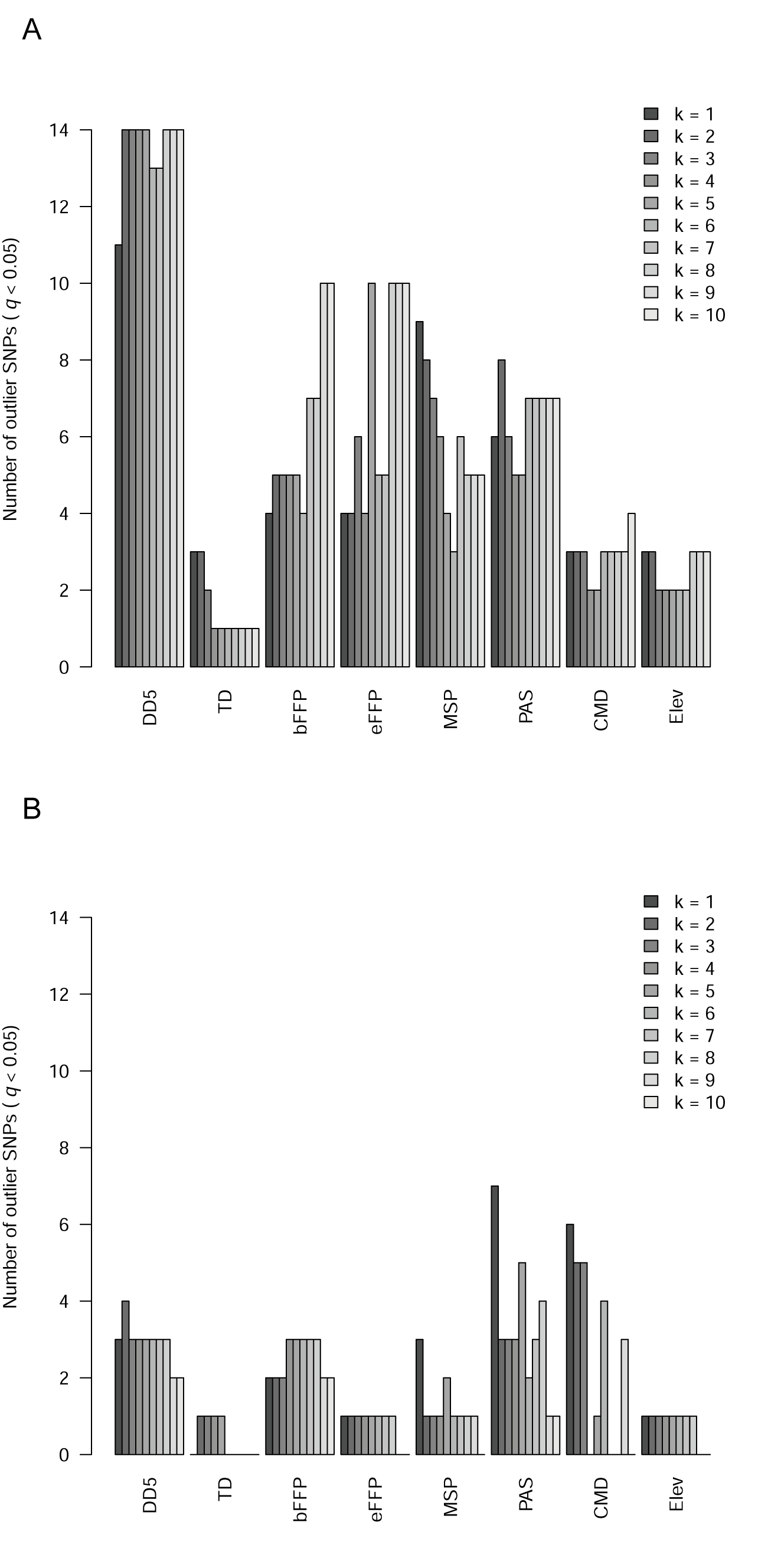


**Figure S3.** (A) *Pinus strobus* and (B) *P. monticola*: number of SNPs associated with each climatic variable (*q* < 0.05) when varying the number of latent factors (*k*) from 1 to 10.


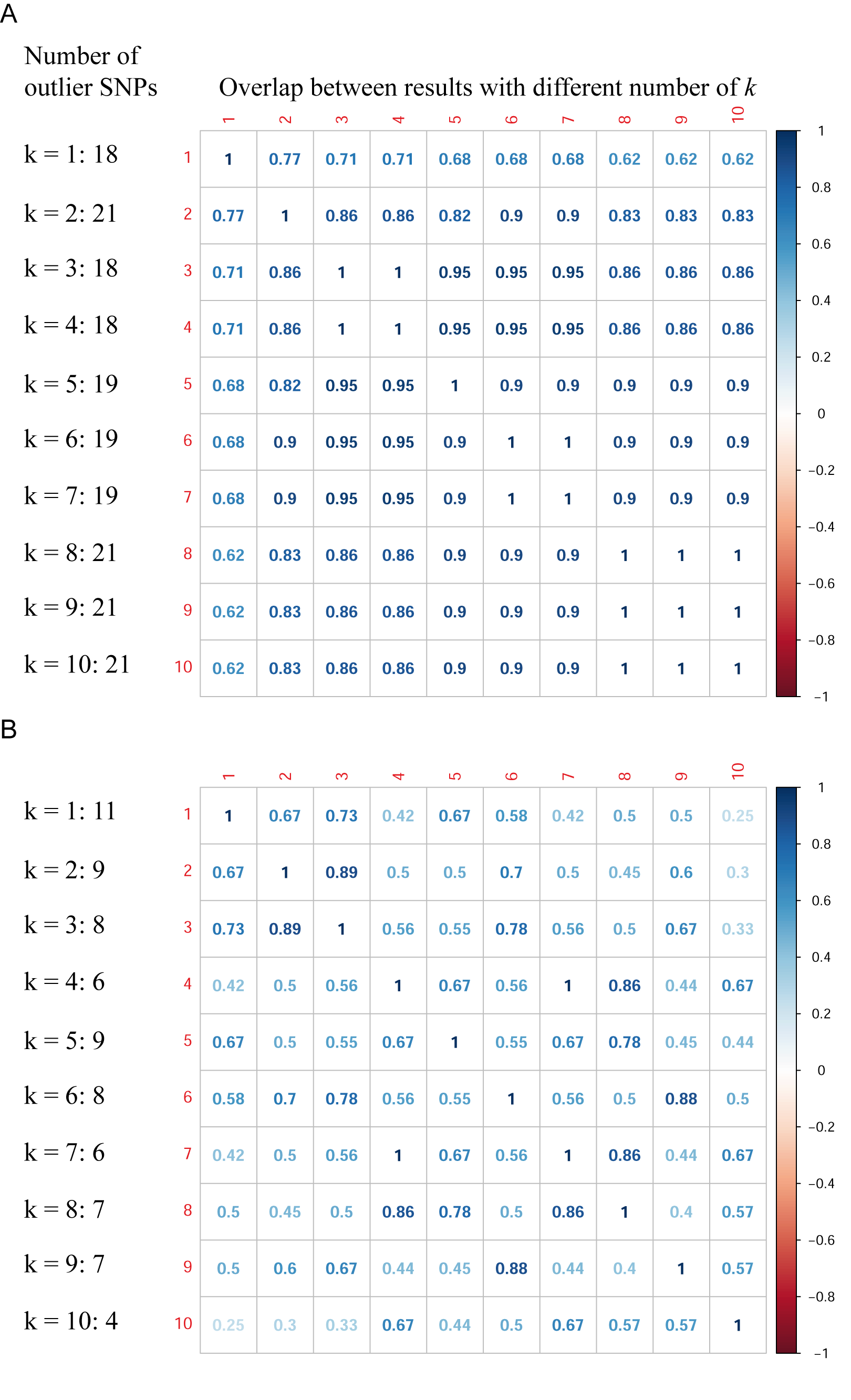


**Figure S4.** (A) *Pinus strobus* and (B) *P. monticola*: overlap between results with different number of latent factor (*k*) calculated as the number of outlier SNPs in common divided by the total number of outlier SNPs).


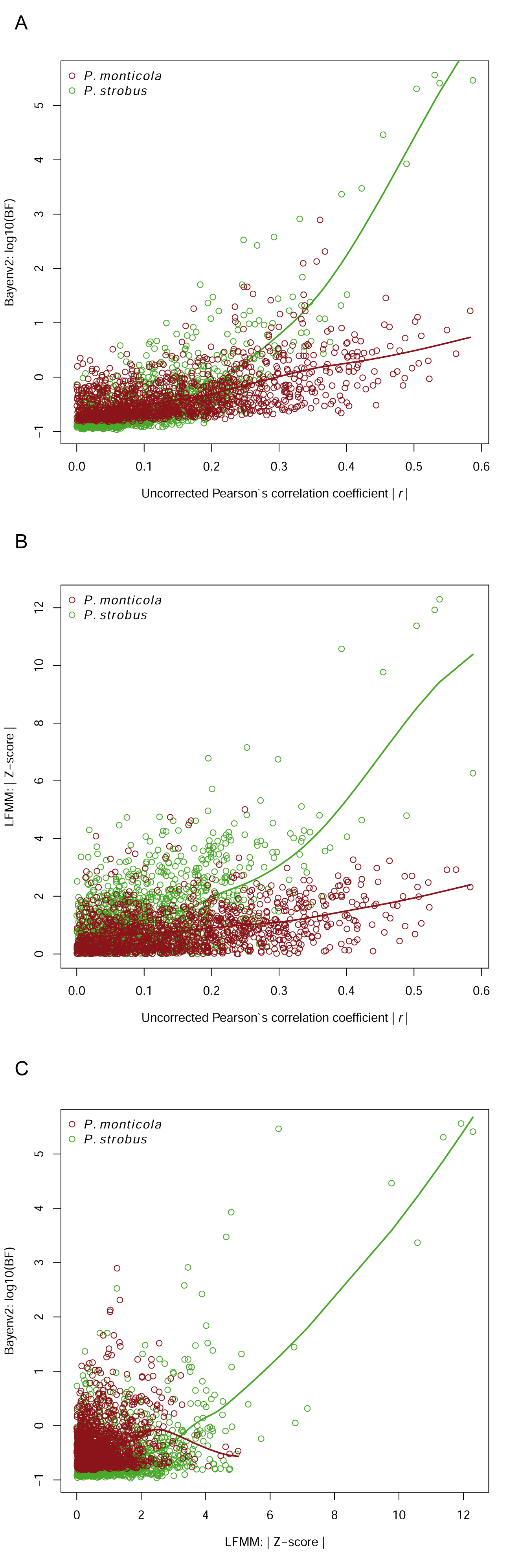


**Figure S5.** Corrections for population structure are smaller for *Pinus strobus* than for *P. monticola*; Relationships between the uncorrected Pearson’s correlation coefficient (*r* in absolute values) calculated for each SNP-climate combination (i.e. not accounting for population structure) and (A) Log10(BF) from Bayenv2 or (B) absolute values of Z-score from LFMM (i.e. accounting for population structure). The Spearman *ρ* correlation between uncorrected *r* and corrected values (Log10(BF) or Z-score) is greater for *P. strobus* than for *P. monticola* (*r* vs. Log10(BF): *P. strobus*: *ρ* = 0.66, *P. monticola*: *ρ* = 0.54; . *r* vs. Z-score: *P. strobus*: *ρ* = 0.42, *P. monticola*: *ρ* = 0.36), indicating that the ranking of SNPs between corrected and uncorrected genetic-environment correlations is more similar in *P. strobus* that in *P. monticola*. (C) Relationship between Log10(BF) and Z-scores (*P. strobus*: *ρ* = 0.23, *P. monticola*: *ρ* = 0.10). A cubic smoothing spline was fitted for each species separately.


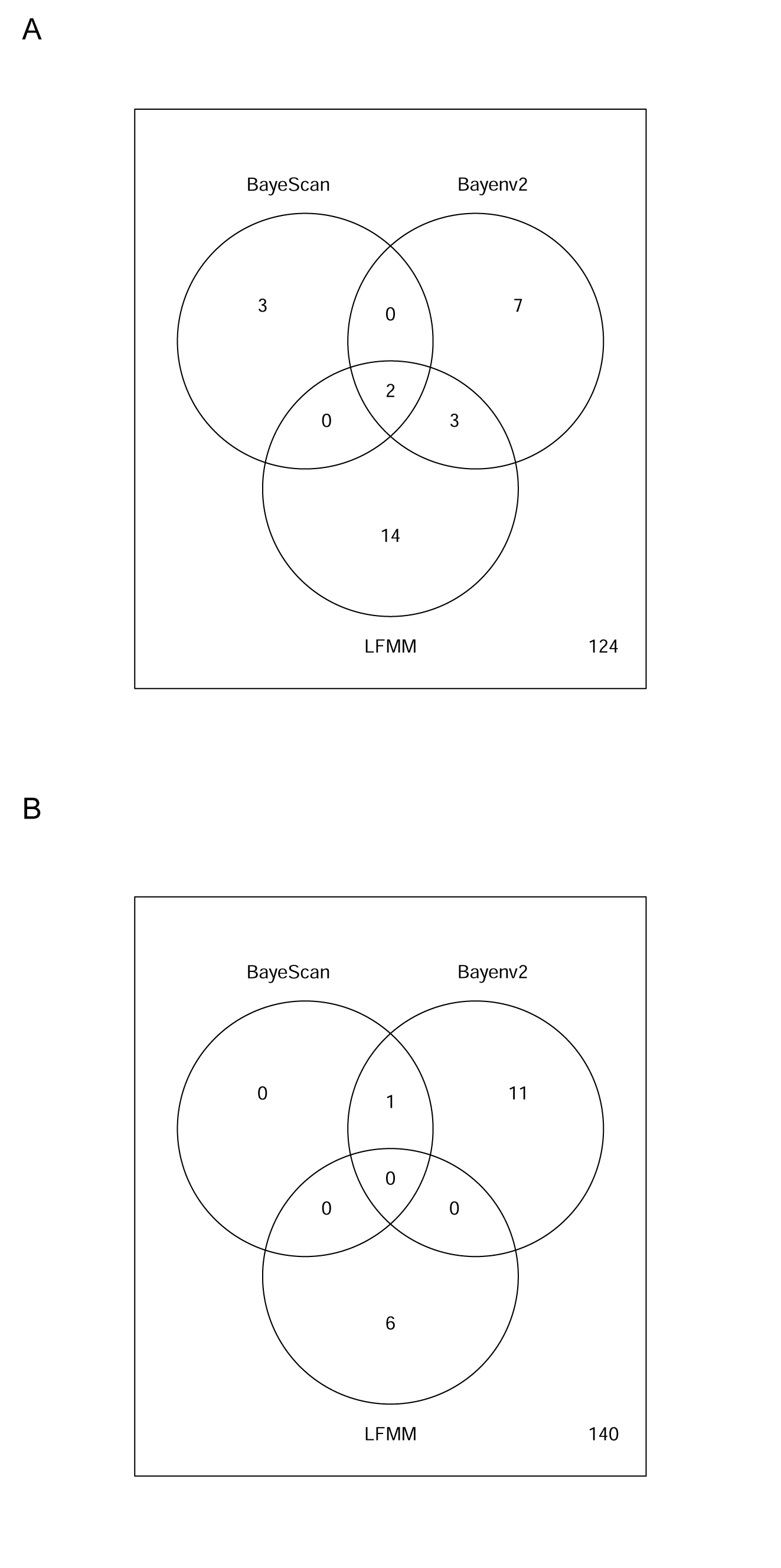


**Figure S6.** (A) *Pinus strobus* and (B) *P. monticola*: overlap in SNPs detected among the three methods (BayeScan, Bayenv2, and LFMM).
